# Associations between degrees of task delegation and adherence to COPD guidelines on spirometry testing in general practice - a national cross-sectional study

**DOI:** 10.1186/s12913-019-4270-3

**Published:** 2019-07-08

**Authors:** Helle Riisgaard, Jette V. Le, Jens Søndergaard, Maria Munch, Loni Ledderer, Line B. Pedersen

**Affiliations:** 1 Research Unit of General Practice Department of Public Health, University of Southern Denmark, J.B. Winsløws Vej 9A, 5000 Odense C, Denmark; 2 Section of Health Promotion and Health Services Department of Public Health, Aarhus University, Bartholins Allé 2 building 1260 225, 8000 Aarhus C, Denmark; 3 DaCHE – Danish Centre of Health Economics Department of Public Health, University of Southern Denmark, J.B. Winsløws Vej 9B, 5000 Odense C, Denmark

**Keywords:** Task delegation, COPD, Spirometry, Guidelines, General practice

## Abstract

**Background:**

The healthcare systems in the western world have in recent years faced major challenges caused by demographic changes and altered patterns of diseases as well as political decisions influencing the organisation of healthcare provisions. General practitioners are encouraged to delegate more clinical tasks to their staff in order to respond to the changing circumstances. Nevertheless, the degree of task delegation varies substantially between general practices, and how these different degrees affect the quality of care for the patients is currently not known. Using chronic obstructive pulmonary disease (COPD) as our case scenario, the aim of the study was to investigate associations between degrees of task delegation in general practice and spirometry testing as a measure of quality of care.

**Methods:**

We carried out a cross-sectional study comprising all general practices in Denmark and patients suffering from chronic obstructive pulmonary disease. General practitioners (GPs) were invited to participate in a survey investigating degrees of task delegation in their clinics. Data were linked to national registers on spirometry testing among patients with COPD. We investigated associations using multilevel mixed-effects logit models and adjusted for practice and patient characteristics.

**Results:**

GPs from 895 practices with staff managing COPD-related tasks responded, and 61,223 COPD patients were linked to these practices. Hereof, 24,685 (40.3%) had a spirometry performed within a year. Patients had a statistically significant higher odds ratio (OR) of having an annual spirometry performed in practices with medium or maximal degrees of task delegation compared to practices with a minimal degree (OR = 1.27 and OR = 1.33, respectively).

**Conclusion:**

Delegating more complex tasks to practice staff implies that COPD-patients are more likely to be treated according to evidence-based recommendations on spirometry testing.

**Electronic supplementary material:**

The online version of this article (10.1186/s12913-019-4270-3) contains supplementary material, which is available to authorized users.

## Background

The healthcare systems in the western world have in recent years faced major challenges caused by changes in demography and the pattern of diseases as well as political decisions influencing the organisation of healthcare provision [1]. Especially the increase in people suffering from chronic conditions and the shift of tasks from secondary to primary care has increased the amount of tasks in general practice [2]. All of this urges the general practitioners (GPs) to rethink the internal working structure in their practices, and it is commonly assumed that delegating more clinical tasks to the practice staff can be an appropriate way of addressing the challenges in primary care [3, 4].

Previous research has shown that healthcare staff has the potential to manage specific clinical tasks with a quality of care equivalent to that of the GPs [4–8]. The evidence is strongest for nurses who have the clinical skills to play an essential part in the management of chronic diseases and complex conditions [9]. For instance, spirometry testing has previously been associated with the presence of a practice nurse and delegation of clinical tasks to the staff [10]. In line with this, one study showed that delegating the treatment of patients with severe hypertension to nurses improved patients’ blood pressure [11], and another study showed an increase of the quality of type 2 diabetes management in general practice [12]. A potential explanation for these findings is that nurses follow guidelines more precisely than physicians and that it influences the quality of care positively. However, a substantial variation in the degree to which GPs choose to delegate tasks to their staff has previously been found [13], and research shows that variation is still present – especially with regard to the complexity of the tasks which the GPs are willing to delegate [14]. Consequently, based on the literature in the area, we hypothesize that higher degrees of task delegation are associated with a higher adherence to guidelines when treating patients with chronic disease.

An example of a highly prevalent chronic condition is chronic obstructive pulmonary disease (COPD), which is a major cause of morbidity and mortality and represents the fourth leading cause of death worldwide [15]. To improve management of COPD, national and international guidelines on evidence-based assessment, diagnosis and treatment have been developed. According to these guidelines, measurement of lung function by spirometry is required to establish the diagnosis and is also an important part of follow-up consultations. Many patients who redeem prescriptions for medication against obstructive lung disease for the first time have not had spirometry performed [16–18], and research has demonstrated gaps in the adherence to recommendations on spirometry testing at COPD follow-up consultations as well [16, 17].

In Denmark, some lack of adherence to COPD guidelines can be observed despite the fact that almost all patients (98%) are listed with a general practice which all have access to a spirometer. To support future decisions and politics on task delegation in general practice and secure a high quality of care for the patients, it is important to explore associations between different degrees of task delegation and the quality of care for patients suffering from COPD as an example of a chronic disease.

## Aim

The objective of this study is to investigate if the degree of task delegation is associated with adherence to guideline recommendations on spirometry testing among COPD patients as an indicator of good quality of care for patients suffering from chronic diseases.

## Methods

### Setting

Denmark has 5.7 m inhabitants and 98% are listed with a specific general practice. General practice in Denmark encompasses approximately 3600 GPs shared among 2200 clinics. The majority of GPs are working in partnership practices [1] and are self-employed based on a collective agreement with the Danish Regions. The main part of the practices employ practice staff, primarily nurses and medical secretaries [19]. According to Danish law the GPs can delegate a wide range of tasks to the staff as long as the staff is provided appropriate education to handle the clinical tasks. It is common to delegate various clinical tasks, also to the medical secretaries, for instance urine tests, drawing of blood samples, and recording of ECGs, while more complex clinical tasks, as for instance consultations related to annual follow-ups, are usually performed by nurses. GPs act as gatekeepers referring to specialists and hospitals. The patients receive all services, including spirometry, for free. Spirometry is primarily performed within the practice by the GP or the practice staff [20], but the GPs can also refer the patients to hospitals or outpatient clinics.

In Denmark, all citizens are assigned a unique personal identification number (CPR) which is registered in the Danish Civil Registration system [21], and every general practice is assigned a unique identification number. These identification numbers are used in national registers and enable researchers to match patients, healthcare services, and general practices [22].

### Study design

The study is part of a large national cross-sectional questionnaire and register-based study covering Danish general practices, the practice staff, and their COPD patients [14, 23–25].

### Questionnaire

In the questionnaire, the GPs were asked to state who were typically undertaking specific COPD-related clinical tasks. The response categories were: “GPs, including GP trainees”, “nurses”, “medical laboratory technicians”, and “secretaries or other employees”. An English version of the complete questionnaire is available as Additional file 1.

The questionnaire was tested in four steps. First, 14 of our academic colleagues assessed and commented on the comprehensibility of the questions. Second, we performed a pilot study including nine GPs testing relevance, acceptability, and feasibility, as well as comprehensibility and completeness. Third, five GPs tested the questionnaire in a qualitative pilot test which was inspired by “The three step test interview” [26]. Fourth, as a specific means of qualifying the organizational aspects of the questionnaire, we performed a focus group interview involving researchers with expertise in this particular field.

The survey covered all general practices in Denmark who had one or more GPs with an email address registered at the Organisation of General Practitioners in Denmark (*n* = 3440). This corresponds to approximately 96% of all GPs in Denmark. For the sampling of practices, see Additional file 2 . On December 4th 2013, the questionnaire was distributed by e-mail, and on January 7th 2014, a reminder was sent out.

The Organisation of Danish GPs provided information on the unique identification number of the practices along with information on the practice form, and GPs’ age and gender.

### Measures

In this study we lean on Niezen’s and Mathijssen’s definition of the procedure of delegating tasks saying that when the care provision shifts from a higher grade person (physician) to a lower grade person (e.g. nurse practitioner), the medical responsibility remains with the higher grade professional [27]. Thus, we define task delegation as: “An intentional transfer of clinical tasks from the GP to another healthcare professional, or to another type of staff member (e.g. secretary), with shorter training and fewer qualifications, while the overall responsibility for the care remains with the GP.”

To identify different degrees of task delegation, we searched the literature for a measure. As no such was found, we developed an algorithm ourselves. It comprised three categories: “minimal degree”, “medium degree”, and “maximal degree” which were used as the explanatory variable in the analyses (see Table 1). The algorithm is desribed and used in another of our studies [14].

**Table 1:** Definition of degrees of task delegation

Degree of task delegation	Definition of the degree of task delegation	Content of delegated tasks
Minimal degree:	No responsibility for assessment in treatment or for decision-making regarding further treatment.	Staff manages laboratory tasks and clinical procedures such as drawing blood samples, measuring blood pressure and performing spirometry.
Medium degree:	Delegated responsibility for assessment in treatment, but no responsibility for decision-making regarding further treatment.	Staff performs more complex tasks such as assessment of functional level, e.g. using an MRC scale, or manages independent consultations, e.g. counseling with regard to smoking cessation or diet and exercise.
Maximal degree:	Delegated responsibility for assessment in treatment and/or decision-making regarding further treatment.	Staff performs highly complex tasks such as assessment of needs for initiating or adjusting COPD medication or assessment of indication for use of antibiotics.

The outcome variable was whether or not COPD patients had an annual spirometry performed. We chose the year 2013, from January 1st to December 31st, as our study period and included spirometries performed in the GP clinics as well as in hospitals or outpatient clinics.

### Register data

**Fig. 1:**
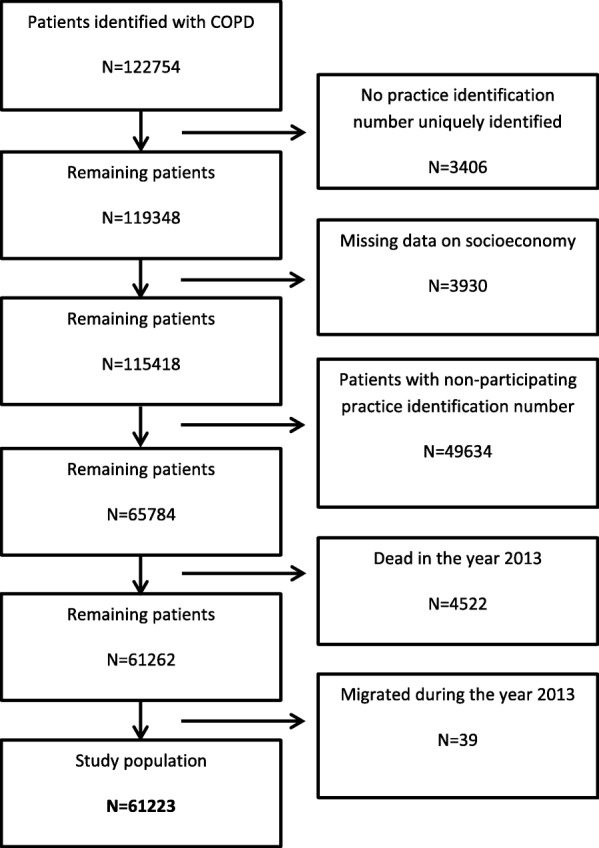
Flow chart of the sampling of COPD patients

To identify patients with COPD, we used the RUKS algorithm, which was developed by the Danish Health Data Board [28]. It defines COPD patients using data from The Danish National Patient Register [29] and The Danish National Prescription Registry [30]. The inclusion criteria of the algorithm were individuals registered with at least one purchase of medication in The Danish National Prescription Register with either a specific indication code for COPD or drugs approved for COPD only and individuals with at least one contact recorded in the National Patient Register with relevant operational or secondary diagnosis for COPD. Patients diagnosed with cystic fibrosis were excluded. To take previous potential registration errors into account, only individuals who had fulfilled the criteria within the previous seven years were included. This limit was chosen due to practical circumstances and deviates from the original RUKS algorithm which defines a 10-year limit (but otherwise uses the same criteria). For the sampling of COPD patients, see Fig. 1.

The identified COPD patients were linked to their regular GP using the unique practice identification number which appears from the registers. This procedure was performed by assessing all the patients’ registered contacts with a GP within the year 2013 and linking to the GP most often visited. If a patient had visited two or more GPs an equal number of times, the GP last consulted was identified as the regular one.

Information on the dates of spirometries was extracted from the National Health Service Register [31] which covers primary care and from the National Patient Register [29] which covers hospitals and outpatient clinics.

Patient characteristics were collected from various demographic and socioeconomic registers, and information on patient comorbidity was extracted from the National Patient Register.

### Data analysis

Multilevel mixed-effects logit models with patients nested within practices were applied to calculate odds ratios with 95% confidence intervals for the associations between task delegation and patients’ odds ratios of having an annual spirometry performed. *P*-values < 0.05 were considered statistically significant.

If GPs within the same practice had different perceptions of the degree of task delegation, we allocated the clinic into the highest degree reported. We thus made this variable a measure of the highest degree of task delegation present in the practice. We used “minimal degree” of delegation as the reference group hypothesizing that higher degrees of delegation are associated with more patients having spirometry performed.

Since previous research has demonstrated associations between specific practice and patient characteristics and spirometry testing [18, 20, 24, 32], we adjusted for these factors in the analyses. Practice characteristics comprised: age and gender of GPs, practice form, status as training practice, and development of standardized processes of care including practice protocols, standard laboratory requisition formulas, and standard recordings in the electronic medical record.

Patient characteristics comprised: age, gender, income, highest attained education, labor market affiliation, and cohabitation status. Further, we chose to adjust for patient comorbidity which was defined according to the Charlson comorbidity index [33].

STATA release 14.1 (StataCorp, College Station, TX, USA) was used for all statistical analyses.

## Results

A total of 3404 of the 3440 invited GPs were eligible for participation, and 1580 responded to the questionnaire corresponding to 46.4%. Of these, 1249 had patients identified with COPD and answered questions essential for the analysis. These respondents belonged to 895 general practices which were included in the study. After having excluded the patients who died or migrated during the study period (the year 2013), patients with missing socioeconomic data, and patients where a unique general practice could not be identified, a total of 61,223 COPD patients were included and linked to a responding practice. See Table 2 for characteristics of the practices. An overview of the entire sampling process of the practices as well as characteristics of patients is available as Additional files 2 and 3, respectively.

**Table 2:** Practice characteristics

	N (%)
Total	895 (100.0)
Degree of delegation	
Minimal	544 (60.8)
Medium	238 (26.6)
Maximal	113 (12.6)
Practice type	
Partnership	568 (63.5)
Single-handed	327 (36.5)
Training practice	
No	291 (32.5)
Yes	604 (67.5)
Mean age of GPs	
45–54	427 (47.7)
55–64	313 (35.0)
< 45	111 (12.4)
> =65	44 (4.9)
Gender of GPs	
Equally mixed	194 (21.7)
All female	200 (22.3)
All male	245 (27.4)
Predominantly female	136 (15.2)
Predominantly male	120 (13.4)

A total of 24,685 (40.3%) COPD patients had a spirometry performed during the observation period. Hereof, 73.3% had it performed in general practice, 7.2% in both general practice and secondary care and 19.6% exclusively in secondary care. In accordance with our hypothesis, the analyses showed that COPD patients had a statistiscally significantly higher odds ratio of having spirometry performed if they were listed with a general practice with medium or maximal degree of delegation compared to a practice with minimal degree of delegation (see Table 3). The odds ratio of COPD patients having a spirometry performed was 33% higher in practices with maximal degree of task delegation and 27% higher in practices with medium degree of task delegation compared to practices with minimal degree of task delegation. The difference between maximal and medium degree was not statistically significant.

**Table 3:** Associations between the degrees of task delegation and COPD patients having an annual spirometry performed

Degree of delegation	N (%)	OR (95% CI)	p-value	OR adj. (95% CI)	p-value
Minimal	33,528 (54.76)	1	–	1	–
Medium	19,027 (31.08)	1.37 (1.25 to 1.49)	< 0.001	1.27 (1.16 to 1.38)	< 0.001
Maximal	8668 (14.16)	1.41 (1.25 to 1.59)	< 0.001	1.33 (1.18 to 1.49)	< 0.001

Adjusted for: Practice characteristics: practice type, age and gender of FPs, status as training practice, and development of standardized processes of care. Patient characteristics: age, gender, income, highest attained education, labor market affiliation, cohabitation status, and comorbidity

## Discussion

Our results show that delegating more complex clinical tasks to practice staff is significantly and positively associated with adherence to clinical practice guidelines on spirometry testing among COPD patients. Our results thereby confirm findings from previous research which indicated that spirometry testing is associated with the presence of a practice nurse and delegation of medical tasks to the staff [10] and add to it by showing that the practices that choose to delegate more complex tasks to their staff also demonstrate a higher adherence to evidence-based guidelines on COPD care.

A potential explanation for the clear-cut association between the two highest degrees of task delegation in general practice and adherence to evidence-based recommendations on spirometry testing among COPD patients may be that delegation of complex tasks from GPs to their staff is often accompanied by a treatment algorithm for assuring safety and quality of care for the patients. Hence, a qualitative study found that providing protocol-based care had the potential to extend the roles of the practice staff, primarily nurses, and influence provision of healthcare positively [34]. These results were supported by a recent survey and register-based study finding that developing standardized processes of care within general practice is associated with adherence to recommendations on spirometry testing among first-time users of medication against obstructive lung diseases [24]. However, even after adjusting for these factors in the analyses, there was still a significantly higher proportion of patients having a spirometry performed in practices with higher degrees of task delegation. This may indicate that high degrees of task delegation are in itself associated with aspects of quality of care for the patients. It may also indicate that GPs and staff who possess better clinical skills are more inclined to engage in higher degrees of task delegation and that they generally follow guidelines to a higher extent and are better at transferring knowledge to their colleagues.

### Strengths and limitations of the study

Since spirometry testing is a process measure on quality of care, it does not allow for conclusions on patient outcomes. Neither does the data provide information on the quality of the performed spirometries, and there is probably some interpractice variation. According to guidelines from the Danish College of General Practitioners [35], healthcare staff who performs spirometry must receive proper training and continuous evaluation. Moreover, the spirometer itself must be calibrated regularly, and quality control has to be ensured. A correctly performed spirometry has important implications for the therapeutic decisions made during follow-up consultations, both regarding choice of pharmacological and non-pharmacological treatments but also in consideration of alternative diagnoses. Further, it is indispensable in identifying rapid decline [36].

Even though spirometry testing is essential in the treatment of COPD [36], there might be cases where it is not possible. For instance, the patient might decline having a spirometry performed for medical reasons such as dementia, frailty, or if the patient has had a tracheotomy. However, these cases are relatively rare and are most likely randomly distributed on the practices in our study. Thus, they can not explain why only 40% of the patients have an annual spirometry performed. Also, since the main criterion in development of the RUKS algorithm [28] is the importance of the patients actually having the disease rather than including all potential cases, it rather underestimates than overestimates the proportion of the patients identified. Thus, the results showing that only 40% of the patients have had a spirometry performed within a year cannot be explained by patients being misclassified as having COPD. The applied method linking patients with their regular GP can have caused an overestimation of the proportion of patients who had a spirometry performed since it was only patients with a contact (of any cause) to a GP that were included. However, this could only have caused a minor distortion since less than 3% of the identified COPD patients did not have a contact with a GP in the year 2013.

We were provided email addresses for 3440 GPs in Denmark from The Organisation of General Practitioners in Denmark, and this number corresponds to around 96% of the entire GP population. Thus, it was a major strength of the study that we were able to invite nearly all Danish GPs to participate in the survey, and furthermore, we obtained a response rate of 46.4%. However, there was an underrepresentation among GPs above 65 years of age (6.6% vs. 10.2%) and males (45.8% compared to 52.2%) as well as GPs in single-handed practices (24.9% vs. 35.6%). Yet, the number of subjects included strengthen the reliability of the findings, and the use of a quality indicator drawn from international recommendations on spirometry testing makes the results relevant and transferable to other countries with a similar organisational setting.

The study was a cross-sectional study reflecting perceptions of the working structure of general practice unlike previous research carried out as an intervention [37] or introduction of new working structures assessed shortly after its implementation [38]. This contributes to strengthening the transferability to everyday practice.

The study period was chosen since it had to be close in time to the distribution of the GP survey. We could have chosen a longer study period but restrained it to one year according to the clinical guidelines for COPD. However, to make sure that a longer study period would not change the results, we performed a sensitivity analysis expanding the period to 18 months. This analysis did not change the overall results, and therefore, the results seem reliable.

### Implications

The finding that delegation of complex clinical tasks to the practice staff is positively associated with provision of evidence-based care has important implications for decisions on the future organisation in general practice. It appears that GPs and practice managers should not be reticent about delegating complex tasks to their staff, at least when taking the quality of care perspective. Also, novel research indicates that this can be done without compromising the job satisfaction of GPs or their staff [14]. More research is, however, needed to investigate associations with quality indicators related to other chronic diseases and preferably aimed at both process and outcome measures such as patient wellbeing or treatment. Also the influence of task delegation on processes and workflow in general practice should be studied in order to maintain the quality of care for the patients. Finally, it would be relevant to investigate the effect of elements regarding task delegation, education, and health protocols on quality of care in an interventional study.

## Conclusion

COPD patients registered with a general practice that delegates more complex clinical tasks to practice staff are more likely to be treated according to evidence-based recommendations on spirometry testing.

## Additional files

Additional file 1: Flow chart of the sampling of practices (PDF 694 kb)

Additional file 2: Patient characteristics (PDF 58 kb)

Additional file 3: Extract of questionnaire for the GPs (PDF 100 kb)

## Data Availability

The data that support the findings of the study are available from the corresponding author on request with permission from the Danish Data Protection Agency.

## Abbreviations

COPD: Chronic Obstructive Pulmonary Disease
GP: General practitioner
