# Supplementary material for: Associations between degrees of task delegation and adherence to COPD guidelines on spirometry testing in general practice - a national cross-sectional study
Source: BMC Health Serv Res. 2019 Jul 8;19:464. doi: 10.1186/s12913-019-4270-3 (PMC6615187; doi:10.1186/s12913-019-4270-3)
Supplement: Supplementary file 1 — Flow chart of the sampling of practices (PDF 694 kb) [file 12913_2019_4270_MOESM1_ESM.pdf]

Thank you for participating in the survey: Organisation in general practice

In the questionnaire we will ask you questions within the following topics:

- Distribution of tasks
- Knowledge implementation
- Job satisfaction

The questions concerning ”distribution of tasks” are specifically related to patients with COPD whereas the remaining questions are general to the work in your practice.

First, we will ask you some questions concerning the structure of your practice.

Which of the practice forms below describe your practice best?
Please mark all that apply

- ❑ Solo practice
- ❑ Partnership practice, including shared practice
- ❑ Collaborative practice, e.g. sharing facilities, clinical staff and/or clinical equipment with another practice (other provider IDs)

On average, how many hours a week do you work in your practice, including work performed at home and attending meetings related to your practice?
Please enter the number of hours

☐

Have you had doctors in training in your clinic within the last year?

- ❑ Yes
- ❑ No

Does your practice employ staff with direct patient contact, e.g. nurse, laboratory technician or secretary?

- ❑ Yes
- ❑ No

How many staff members are employed in your practice?
Please enter a number for each category

Nurse(s) ☐

Laboratory technician(s) ☐

Secretary/Secretaries and/or other staff members ☐

Which of the following tasks does your staff perform?
Please mark all that apply

- ❑ Secretarial duties (e.g. answering the telephone, reception desk or renewing prescriptions)
- ❑ Laboratory work (e.g. performing ECG, urine analysis, or drawing blood samples
- ❑ Clinical procedures (e.g. rinsing the external ear, treating warts, or administering vaccinations)
- ❑ Follow-up care of chronic diseases (e.g. COPD, diabetes or hypertension)

How many staff members in your practice undertake tasks regarding management of patients with COPD, including laboratory work, performing clinical procedures and/or independent consultations?
Please enter the number below

☐

If you have any comments to the questions in this section, you may write them in the box below:

☐

We will now ask you some questions, which concern task delegation regarding management of patients with COPD in your practice

The questions are about, who undertakes various tasks in each of the following types of consultations with COPD patients:

- Diagnosis
- Annual follow-up
- Semiannual and quarterly follow-up
- Exacerbations

Who is **typically** undertaking the following task regarding **diagnosis** of patients with COPD in your practice?

Select one or more answers

	GP, including GP trainee	Nurse	Medical laboratory technician	Secretary or other staff member	Not performed/ Performed elsewhere	Do not know
Performing spirometry	❑	❑	❑	❑	❑	❑
Recording patient's medical history	❑	❑	❑	❑	❑	❑
Drawing blood samples	❑	❑	❑	❑	❑	❑
Measuring oxygen saturation	❑	❑	❑	❑	❑	❑
Instructing in inhalation technique	❑	❑	❑	❑	❑	❑
Measuring inspiratory flow	❑	❑	❑	❑	❑	❑
Assessment of needs for initiating or adjusting COPD medication	❑	❑	❑	❑	❑	❑

Assessment of functional level – e.g. MRC scale	☐	☐	☐	☐	☐	☐
Assessment of quality of life, e.g. CAT-score	☐	☐	☐	☐	☐	☐
Assessment of sputum, e.g. according to the patient's description	☐	☐	☐	☐	☐	☐
Counseling with regard to self-care	☐	☐	☐	☐	☐	☐
Measuring blood pressure	☐	☐	☐	☐	☐	☐

Who is **typically** undertaking the following task regarding **annual follow-up** of patients with COPD in your practice?
Select one or more answers

	GP, including GP trainee	Nurse	Medical laboratory technician	Secretary or other staff member	Not performed/ Performed elsewhere	Do not know
Performing spirometry	☐	☐	☐	☐	☐	☐
Reviewing history of disease progression, e.g. number of exacerbations within the last year	☐	☐	☐	☐	☐	☐
Drawing blood samples	☐	☐	☐	☐	☐	☐
Measuring oxygen	☐	☐	☐	☐	☐	☐

saturation						
Instructing in inhalation technique	❑	❑	❑	❑	❑	❑
Measuring inspiratory flow	❑	❑	❑	❑	❑	❑
Reviewing patients’ COPD medication	❑	❑	❑	❑	❑	❑
Assessment of functional level, e.g. MRC scale	❑	❑	❑	❑	❑	❑
Assessment of quality of life, e.g. CAT-score	❑	❑	❑	❑	❑	❑
Assessment of sputum, e.g. according to the patient’s description	❑	❑	❑	❑	❑	❑
Counseling with regard to self-care	❑	❑	❑	❑	❑	❑
Counseling with regard to vaccination against influenza and pneumonia	❑	❑	❑	❑	❑	❑
Counseling with regard to smoking cessation	❑	❑	❑	❑	❑	❑
Counseling with regard to diet and exercise	❑	❑	❑	❑	❑	❑
Measuring blood pressure	❑	❑	❑	❑	❑	❑

Performing echocardiography	❑	❑	❑	❑	❑	❑

Who is **typically** undertaking the following task regarding **semiannual and quarterly follow-up** of patients with COPD in your practice?
Select one or more answers

	GP, including GP trainee	Nurse	Medical laboratory technician	Secretary or other staff member	Not performed/ Performed elsewhere	Do not know
Performing spirometry	❑	❑	❑	❑	❑	❑
Drawing blood samples	❑	❑	❑	❑	❑	❑
Measuring oxygen saturation	❑	❑	❑	❑	❑	❑
Instructing in inhalation technique	❑	❑	❑	❑	❑	❑
Measuring inspiratory flow	❑	❑	❑	❑	❑	❑
Assessment of needs for initiating or adjusting COPD medication	❑	❑	❑	❑	❑	❑
Assessment of functional level, e.g. MRC scale	❑	❑	❑	❑	❑	❑
Assessment of quality of life, e.g. CAT-score	❑	❑	❑	❑	❑	❑
Assessment of sputum, e.g.	❑	❑	❑	❑	❑	❑

according to the patient's description						
Counseling with regard to self-care	❑	❑	❑	❑	❑	❑
Counseling with regard to vaccination against influenza and pneumonia	❑	❑	❑	❑	❑	❑
Counseling with regard to smoking cessation	❑	❑	❑	❑	❑	❑
Counseling with regard to diet and exercise	❑	❑	❑	❑	❑	❑
Measuring blood pressure	❑	❑	❑	❑	❑	❑

Who is **typically** undertaking the following task regarding **exacerbations** in patients with COPD in your practice?
Select one or more answers

	GP, including GP trainee	Nurse	Medical laboratory technician	Secretary or other staff member	Not performed/ Performed elsewhere	Do not know
Performing stethoscopy	❑	❑	❑	❑	❑	❑
Drawing blood samples, e.g. CRP	❑	❑	❑	❑	❑	❑
Measuring oxygen saturation	❑	❑	❑	❑	❑	❑
Assessment of needs for initiating or adjusting COPD	❑	❑	❑	❑	❑	❑

medication						
Assessment of sputum, e.g. according to the patient’s description	❑	❑	❑	❑	❑	❑
Assessment of indication for use of antibiotics	❑	❑	❑	❑	❑	❑
Assessment of indication for use of prednisolone	❑	❑	❑	❑	❑	❑
Counseling with regard to self-care	❑	❑	❑	❑	❑	❑
