# Supplementary figures and images for: Associations between degrees of task delegation and adherence to COPD guidelines on spirometry testing in general practice - a national cross-sectional study
Source: BMC Health Serv Res. 2019 Jul 8;19:464. doi: 10.1186/s12913-019-4270-3 (PMC6615187; doi:10.1186/s12913-019-4270-3)

Flow chart of the sampling of practices

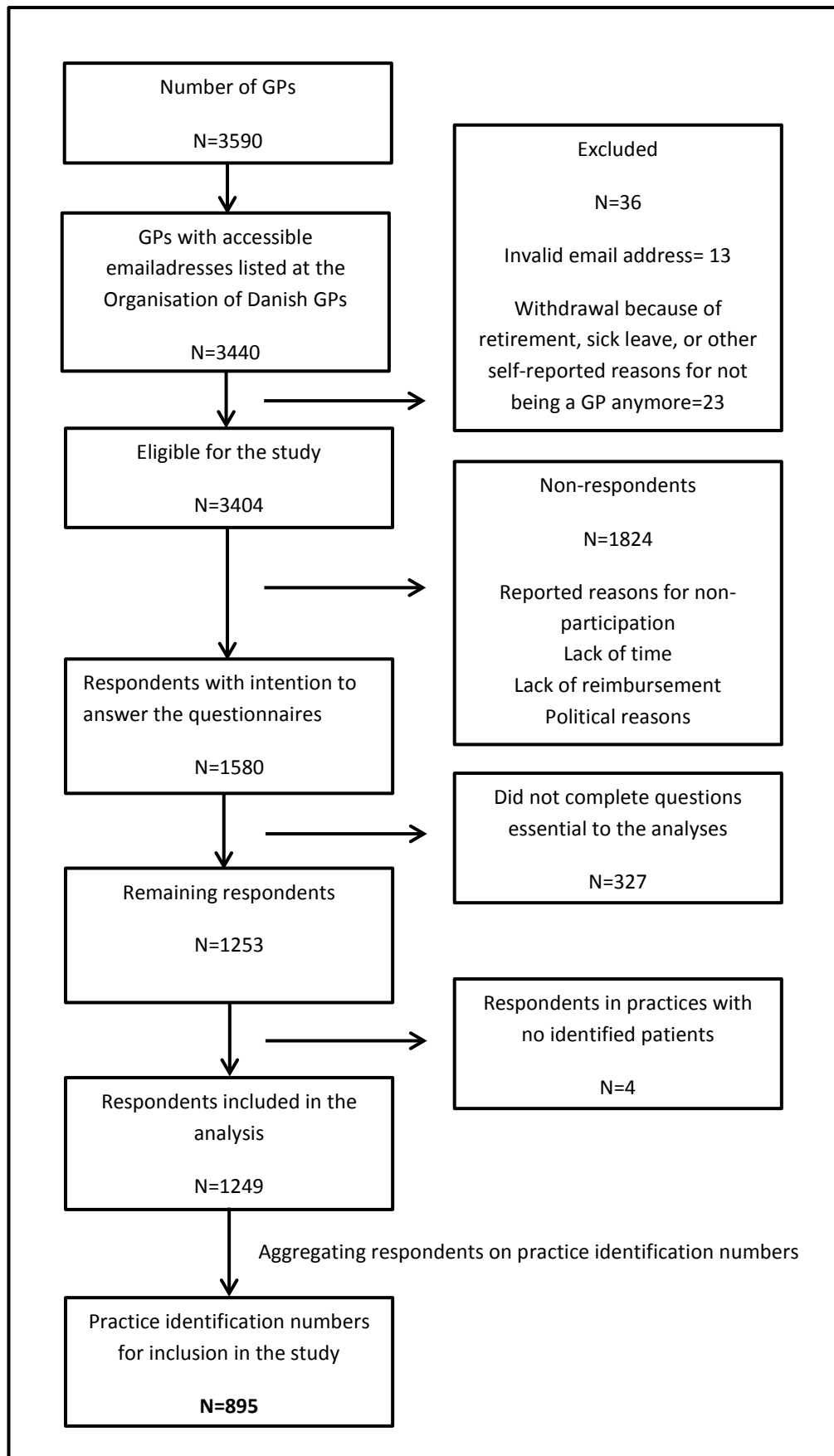

Supplement: Supplementary file 2 — Patient characteristics (PDF 58 kb) [file 12913_2019_4270_MOESM2_ESM.pdf]
