# Supplementary material for: Associations between degrees of task delegation and adherence to COPD guidelines on spirometry testing in general practice - a national cross-sectional study
Source: BMC Health Serv Res. 2019 Jul 8;19:464. doi: 10.1186/s12913-019-4270-3 (PMC6615187; doi:10.1186/s12913-019-4270-3)
Supplement: Supplementary file 3 — Extract of questionnaire for the GPs (PDF 100 kb) [file 12913_2019_4270_MOESM3_ESM.pdf]

Patient characteristics

	N (%)
**Total**	61,223 (100.0)
**Comorbidity (Charlson index)**	
0	12,587 (20.6)
1	21,109 (34.5)
2	11,020 (18.0)
3	16,507 (27.0)
**Cohabitation status**	
Living alone	26,922 (44.0)
Married/cohabitating	34,301 (56.0)
**Household income**	
Lowest quartile	19,091 (31.2)
Middle quartiles	34,098 (55.7)
Highest quartile	8,034 (13.1)
**Education**	
<10 years (basic school)	31,467 (51.4)
10-12 years	22,158 (36.2)
>12 years	7,598 (12.4)
**Labour market affiliation**	
Working	11,350 (18.5)
Retired	38,612 (63.1)
Unemployed	11,261 (18.4)
**Gender**	
Male	27,020 (44.1)
Female	34,203 (55.9)
**Age**	
Mean age (std.)	67.0 (12.1)
